# Adenosquamous carcinoma development as a recurrence of squamous cell carcinoma in the oral floor: A case report

**DOI:** 10.1097/MD.0000000000017688

**Published:** 2019-10-25

**Authors:** Takanori Eguchi, Akihiko Basugi, Ikuyo Kanai, Yukinaga Miyata, Takamasa Suzuki, Yoshiki Hamada

**Affiliations:** a Department of Oral and Maxillofacial Surgery, School of Dental Medicine, Tsurumi University, Yokohama; b Department of Oral and Maxillofacial Surgery; c Department of Pathology, Toshiba Rinkan Hospital, Sagamihara, Japan.

**Keywords:** cancer origin, histogenesis, head and neck cancer, oral cancer, recurrence

## Abstract

**Rationale:**

Oral adenosquamous carcinoma (ASC) is rare and its origins are controversial. We here present a patient with oral ASC that developed after surgery for oral squamous cell carcinoma (SCC).

**Patient concerns:**

A 70-year-old man with SCC on the oral floor underwent surgical resection. However, the enlarged ulcer presented on the oral floor 9 month after surgery.

**Diagnoses:**

The biopsy of the ulcer revealed a SCC. Imaging examinations detected enhancement of a large lesion expanded to the tongue, but no evidence of regional lymph node or distant metastasis was shown. Based on these results, local recurrence of the cancer was diagnosed (cT4aN0M0).

**Interventions:**

The surgery for the recurrent tumor was performed.

**Outcomes:**

The pathological examination of the surgical specimen indicated recurrent tumor was ASC. Thus, histopathological and immunohistochemical analyses of both the initial SCC and the subsequent ASC were performed in an attempt to explore the origin of the ASC. As the results, pathological review of both tumors suggested the subsequent ASC was developed from the tumor cells with adenoid phenotype in the initial SCC.

**Lessons:**

This report suggests that the oral ASC was origin from the oral SCC, which can contribute to new knowledge for pathogenesis of oral cancer.

## 1 Introduction

Adenosquamous carcinoma (ASC) is an aggressive malignant epithelial neoplasm that rarely occurs in the oral cavity.^[1]^ ASC reportedly develops more than twice as frequently in men than in women.^[2]^ The prognosis is poor because of the high incidence of regional lymph node and distant metastasis, and the 5-year survival rate is 13% to 50%.^[1]^

Histopathologically, ASC consists of both squamous cell carcinoma (SCC) and adenocarcinoma (AC) components.^[1]^ Although the term “adeno-" is included in its name, the World Health Organization (WHO) classifies ASC as a rare variant of SCC but not as an independent entity.^[1]^ However, the histogenesis of ASC is poorly understood because of its rarity.

In this report, we present an ASC that developed after surgery and chemotherapy for a SCC of the oral cavity. Histopathological and immunohistochemical analyses of both the initial SCC and subsequent recurrent ASC were performed to explore the origin of the recurrent ASC.

## 2 Case report

### 2.1 Clinical course

**Figure 1:**
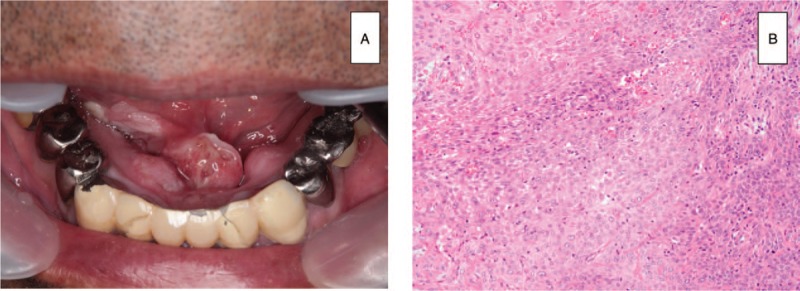
Initial SCC. (A) Image of a painful 26 mm × 17 mm nodule with induration located in the oral floor. (B) Photomicrograph showing histopathological features of SCC (HE stain).

A 70-year-old Japanese man was referred to our clinic because of a persistent painful nodule in the floor of the oral cavity. His medical history included controlled hypertension and diabetes mellitus, and he had been a habitual smoker. Clinical examination revealed a 26 × 17 mm-sized painful nodule with induration on the oral floor (Fig. 1A). Contrast-enhanced-computed tomography (CECT) imaging showed an enhanced lesion, and ^18^F-fluorodeoxyglucose-positron emission tomography (FDG-PET) detected a maximum standardized uptake value (SUV max) of 7.3 in the oral floor without abnormal uptake in other regions such as the cervical lymph nodes and lung. An incisional biopsy resulted in a diagnosis of SCC (cT2N0M0).

One month later, he underwent ablation of the oral floor cancer with marginal resection of the adjacent mandible under general anesthesia. At that time, the sublingual glands and Wharton ducts were also resected bilaterally to ensure an adequate margin, and the openings of both ducts were relocated posteriorly. Histopathological examination of the surgical specimen showed a moderately differentiated SCC with lymphatic, perineural, and vascular invasion (Fig. 1B). The surgical margin as well as the sublingual glands and the resected bone were free of tumor. However, the distance from the inferior surgical margin to the resected genioglossus muscles was less than 10 mm. S-1 (tegafur/gimeracil/oteracil) was thus prescribed from postoperative day 27 as adjuvant chemotherapy (cycles of 100–120 mg/day for 2 weeks followed by a 2-week rest for 1 year).

**Figure 2:**
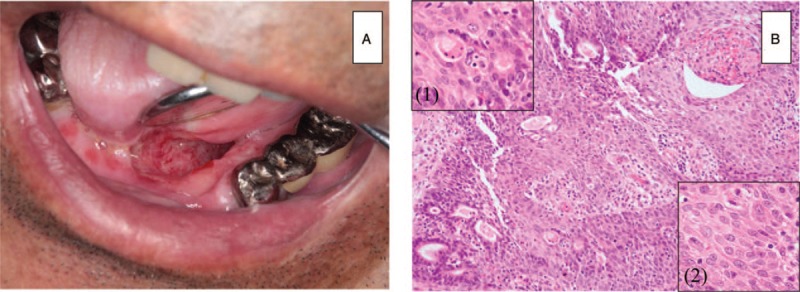
Subsequent ASC. (A) Photograph of the ulcer in the oral floor prior to the second surgery. (B) Photomicrograph showing histopathological features of ASC (HE stain). The insert shows the AC (1) and SCC components (2).

Two cytological examinations of the operative ulcer on the oral floor revealed no malignancy at 3- and 6-month follow-ups. Additionally, imaging examinations showed no evidence of recurrence or metastasis of the cancer. However, the ulcer was enlarged 9 months after the surgery (Fig. 2A), and a malignancy was highly suspected by cytological examination. Subsequent incisional biopsy revealed a moderately differentiated SCC. CECT imaging detected enhancement of a lesion from the oral floor to the deep layers of the tongue bilaterally, but there was no evidence of regional lymph node or distant metastasis. These results indicated local recurrence of the cancer (cT4aN0M0), and subtotal glossectomy with immediate reconstruction using a forearm flap, bilateral neck dissection, and tracheostomy were performed under general anesthesia. Histopathological examination of the surgical specimen showed that most of the tumor consisted of moderate and poorly differentiated SCC, and tumor cells with a glandular structure were also revealed (Fig. 2B). In addition, cervical lymph node metastases in the right level 2 and left levels 1 and 3 were detected, resulting in a diagnosis of ASC with bilateral lymph node metastasis (pT4aN2cM0). Consequently, concurrent chemoradiation therapy (cisplatin and irradiation) was performed from postoperative day 40 as adjuvant therapy. However, the initial dose of cisplatin caused acute renal impairment, and the adjuvant therapy was thus discontinued. Thirteen months after the original surgery, FDG-PET indicated local recurrence and multiple lymph node metastases again, and the patient died of respiratory obstruction caused by the tumor.

### 2.2 Histopathological and immunohistochemical (IHC) analyses

Hematoxylin and eosin [HE]-stained samples of formalin-fixed and paraffin-embedded tissues of the surgical specimens from both the initial and the recurrent tumors were carefully reviewed, and the tumors were subjected to IHC examination. For IHC analyses, cytokeratins 7 and 20 (CK7, CK20), carcinoembryonic antigen (CEA), p53, and p40 were assessed using a labeled streptavidin–biotin immunostaining method. The staining results were classified as positive (+) or negative (−).

Histopathological examination of the initial tumor showed a moderately differentiated SCC with its deep part diffusely invading adjacent tissue in a cord-like manner. The tumor cells were CK7^−^, CK20^−^, and p40^+^. Around 80% of the tumor cells were strongly positive for p53, arguing its coding gene (*TP53*) had undergone mutational events. Interestingly, some of the tumor cells also expressed CEA, although the staining was somewhat weak, implying the possibility of pre-existing tumor cells with a potential adenoid phenotype in the initial SCC.

Careful examination of HE-stained sections of the recurrent tumor also showed that its major component was moderately to poorly differentiated SCC; however, a definite AC component with formation of glandular structures was also revealed, and there were also areas in which AC and SCC components intermingled. Thus, the recurrent tumor was histopathologically diagnosed as ASC. As to the IHC findings, tumor cells in the SCC component were basically CEA^−^, CK7^−^, CK20^−^, and p40^+^. Interestingly, some tumor cells were CEA^+^ and CK7^+^, which indicated the existence of tumor cells with a possible adenoid phenotype in the SCC components. On the other hand, the AC component with glandular structures was CEA^+^, CK7^+^, and CK20^−^. Surprisingly, a few tumor cells in the glandular structures were p40^+^, indicating the existence of tumor cells with a potential squamous cell phenotype in the ASC components. Additionally, the distribution and intensity of p53 positivity did not differ between the AC and SCC components, suggesting a common genetic event for *TP53* in both.

**Figure 3:**
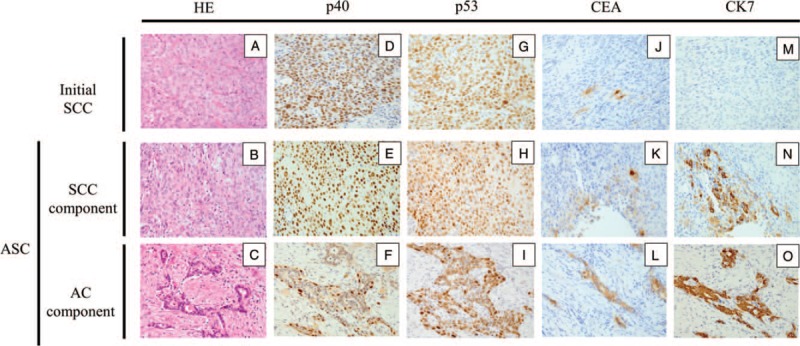
Histopathological and immunohistochemical (IHC) analyses. Photomicrographs showing immunohistopathological findings. (A–C) HE stain, (D–F) p40, (G–I) p53, (J–L) CEA, and (M–O) CK7. (A) Moderately differentiated SCC in the initial SCC. (B) Poorly differentiated SCC in the SCC component. (C) Well-differentiated glandular structures in the AC component. (D) p40^+^ cell in the initial SCC. (E) p40^+^ cells in the SCC component. (F) p40^+^ cells in the AC component. (G) p53^+^ cells in the initial SCC. (H) p53^+^ cells in the SCC component. (I) p53^+^ cells in the AC component. (J) CEA^+^ cells in the initial SCC. (K) CEA^+^ cells in the SCC component. (L) CEA^+^ cells in the AC component. (M) CK7^−^ cells in the initial SCC. (N) CK7^+^ cells in the SCC component. (O) CK7^+^ cells in the AC component.

The histopathological and IHC findings in both the initial SCC and subsequent ASC are summarized in Figure 3A–O and Table 1.

**Table 1:**
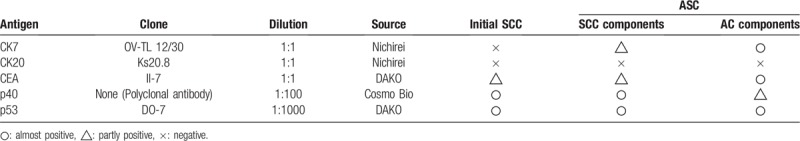
Summary of immunohistochemical analysis of both initial SCC and ASC.

## 3 Discussion

ASC is histopathologically characterized by a mixture of distinct components consisting of AC and SCC.^[1]^ Although these 2 components are very close in proximity and are generally distinct, mixed areas, or areas of confluency may also exist.^[3]^ The SCC component is morphologically identical to the usual SCC, and it may show varying grades of differentiation.^[4]^ The AC component has tubular/ductal morphology with intraluminal or intracellular mucin^[1]^ and is usually found in the deeper areas of the tumor, whereas the SCC component appears in the superficial areas.^[1]^ Thus, incisional biopsy alone is generally difficult for diagnosis of ASC. Yoshimura et al reported an incorrect diagnosis rate based on incisional biopsy of 68.5%, and the most frequent misdiagnosis from pretreatment biopsy was SCC (36.5%).^[5]^ Our case was also diagnosed as SCC in the pretreatment biopsy. We therefore consider that incisional biopsy with depth is important for an accurate diagnosis.

Immunohistochemical examinations support the accurate diagnosis of ASC. AC components express CK7 and often CEA,^[6,7]^ but are negative for CK20. SCC components express p63 or p40,^[6,7]^ while p53 overexpression is often observed in both components.^[6]^ Although adenoid (acantholytic) SCC may mimic ASC due to the acantholysis (gland-like space) of the tumor cells, it is easily diagnosed because the tumor does not express CK7 and CEA.^[6]^ Basaloid SCC and conventional SCC can also be diagnosed by the presence of tumor cells without a glandular malignant component.^[1]^ On the other hand, differential diagnosis of mucoepidermoid carcinoma is difficult, because immunohistochemical analysis cannot generally distinguish it from ASC. Mumcoepidermoid carcinoma characteristically has intermediate or transitional cells, but these are lacking in ASC.^[1,6]^ Based on these evidences, we were able to diagnose ASC in our current case.

The histogenesis of oral ASC is controversial, and there is a dogma as to whether these cancers arise from salivary gland tissue or superficial squamous epithelium.^[6,8,9]^ In the presented case, IHC analyses of the ASC revealed that the SCC components had an adenoid phenotype, and the AC components had a squamous cell phenotype. This unique finding suggests that cells with the adenoid phenotype in the SCC component had the potential to differentiate to AC, and the squamous cell phenotype cells in the AC component originated from the SCC. This is because the AC components formed well-differentiated glandular structures, whereas the SCC components were poorly differentiated; a cell generally matures from poorly- to well-differentiated.^[10]^ Moreover, the distribution of p53 positivity was common between the SCC and AC components, which implies that the common *TP53* mutations occurred in both components. In fact, Kanazawa et al reported that the AC component originates from SCC in lung ASC, because the distributions and mutations of *TP53* were common in both the SCC and AC components, and the expression of SCC-related antigen in the AC component was higher than in conventional lung AC.^[11]^ Based on these findings, the AC components developed from within the SCC components, which suggests that oral ASC originated from SCC.

Our theory that the oral ASC originated from SCC in our case is supported by the IHC findings of the initial SCC. The initial SCC included CEA^+^ cells; CEA is a typically expressed by endodermally-derived tissues and adenocarcinomas.^[12]^ This finding indicates that the initial SCC had tumor cells with the adenoid phenotype. Moreover, the distribution of p53 positivity was the same in the initial SCC and in the subsequent ASC, which indicates that ASC was an immunohistopathological recurrence of the initial SCC. Thus, we consider that the CEA^+^ cells in the initial SCC differentiated to AC, resulting in ASC.

The prognosis of ASC in the head and neck is very poor because a treatment strategy, especially adjuvant therapy, has not yet been established. Meng et al reported that patients with cervical ASC experience relatively better survival benefits from adjuvant radiotherapy.^[13]^ In our case, adjuvant therapy was not completed because of the severe adverse effects of cisplatin, and local recurrence and multiple lymph node metastases occurred within 2 months after surgery. Although further research should be conducted to establish treatment strategies for ASC in head and neck cancer, we postulate that surgery alone may not suppress ASC because of regional lymph and distant metastasis.

We diagnosed that the ASC was clinically a recurrence of the initial SCC because the imaging examinations demonstrated no evidence that the oral lesion was distant metastasis from another organ. Additionally, the excised ulcer on the oral floor did not heal, implying the residual initial SCC was in this location. Although we cannot completely rule out that the ASC was not a *de novo* cancer, all the above supports the notion that the ASC was a recurrence of the SCC.

## 4 Conclusions

The oral ASC in this case developed from the initial SCC via differentiation of SCC cells with an adenoid phenotype into AC, suggesting that the oral ASC originated from the oral SCC. Therefore, oral ASC can arise from anywhere in the oral region if SCC cells with this adenoid phenotype are present. This could explain the dogma as to whether ASC arises from salivary gland tissue or superficial squamous epithelium.

## Acknowledgments

We thank Dr Trish Reynolds, MBBS, FRACP, and H. Nikki March, PhD, from Edanz Group (www.edanzediting.com/ac) for editing a draft of this manuscript.

## Author contributions

**Conceptualization:** Takanori Eguchi.

**Data curation:** Takanori Eguchi, Akihiko Basugi, Ikuyo Kanai, Yukinaga Miyata.

**Formal analysis:** Takamasa Suzuki.

**Investigation:** Takanori Eguchi, Takamasa Suzuki.

**Resources:** Takanori Eguchi.

**Writing – original draft:** Takanori Eguchi.

**Writing – review & editing:** Takanori Eguchi, Yoshiki Hamada.

Takanori Eguchi orcid: 0000-0003-4608-1347.
